# Affective and cognitive empathy in adolescents with autism spectrum disorder

**DOI:** 10.3389/fnhum.2014.00791

**Published:** 2014-10-07

**Authors:** Monica Mazza, Maria C. Pino, Melania Mariano, Daniela Tempesta, Michele Ferrara, Domenico De Berardis, Francesco Masedu, Marco Valenti

**Affiliations:** ^1^Department of Life, Health and Environmental Sciences, University of L’AquilaL’Aquila, Italy; ^2^Psychiatric Service of Diagnosis and Treatment, Department of Mental Health, G. Mazzini HospitalTeramo, Italy; ^3^Department of Applied Clinical Sciences and Biotechnology, Section of Clinical Epidemiology and Environmental Medicine University of L’AquilaL’Aquila, Italy; ^4^Reference Regional Centre for Autism, Abruzzo Region Health SystemL’Aquila, Italy

**Keywords:** adolescents, autistic spectrum disorder (ASD), affective empathy, cognitive empathy, experience sharing, mentalizing

## Abstract

The broad construct of empathy incorporates both cognitive and affective dimensions. Recent evidence suggests that the subjects with autistic spectrum disorder (ASD) show a significant impairment in empathic ability. The aim of this study was to evaluate the cognitive and affective components of empathy in adolescents with ASD compared to controls. Fifteen adolescents with ASD and 15 controls underwent paper and pencil measures and a computerized Multifaceted Empathy Test. All measures were divided into mentalizing and experience sharing abilities. Adolescents with ASD compared to controls showed deficits in all mentalizing measures: they were incapable of interpreting and understanding the mental and emotional states of other people. Instead, in the sharing experience measures, the adolescents with ASD were able to empathize with the emotional experience of other people when they express emotions with positive valence, but were not able to do so when the emotional valence is negative. These results were confirmed by the computerized task. In conclusion, our results suggest that adolescents with ASD show a difficulty in cognitive empathy, whereas the deficit in affective empathy is specific for the negative emotional valence.

## INTRODUCTION

Autistic Spectrum Disorder (ASD) is a triad of qualitative impairments in social interaction, communication and restricted, repetitive, and stereotyped behaviors (American Psychiatric Association, 2000). An important feature of the proposed criteria in DSM-5 for ASD is a change from three (autistic triad) to two domains: “social/communication deficits” and “fixated and repetitive pattern of behavior” (Wilkinson, 2012). These difficulties often make it very hard for people with ASD to be successful members of society and can present very serious challenges to parents, teachers, and other professionals. Major difficulties in social interaction have been a defining feature of individuals with autism (Fletcher-Watson et al., 2014).

People with ASD often show an impaired comprehension of other people’s mental states, such as thoughts, beliefs, and intentions (Frith and Happé, 1994; Frith and Frith, 2003; Jones et al., 2010; Gaigg, 2012; Schwenck et al., 2012). Recent studies showed that subjects with ASD have not only a difficulty in attributing another person’s mental state but also in the capacity to respond to another person’s mental state with an appropriate emotion (Sucksmith et al., 2013). These abilities seem to be involved in the multifaceted construct of empathy (Zaki and Ochsner, 2012). In agreement with recent literature (Jones et al., 2010; Baron-Cohen, 2011; Dziobek et al., 2011; Schwenck et al., 2012; Zaki and Ochsner, 2012) empathy should no longer be considered as a unitary concept, instead it comprises at least two components (Singer, 2006; Decety and Meyer, 2008; Dziobek et al., 2008). In fact, empathy includes the ability to understand what others are thinking or feeling, without necessarily “resonating” with that feeling state (cognitive empathy) and the ability to emotionally “resonate” with other people’s feelings while understanding that they are distinct from one’s own (affective empathy; Jones et al., 2010; Schwenck et al., 2012). The cognitive dimension of empathy requires complex cognitive functions, including perspective-taking and mentalizing (Shamay-Tsoory et al., 2002, 2009; Shamay-Tsoory, 2011; Zaki and Ochsner, 2012), whereas affective empathy includes experience sharing of other persons’ internal states (Zaki and Ochsner, 2012). Emotional contagion is a precursor of affective empathy, whereby embodiment entails the forming of a representation of the other person’s feelings, and thereby sharing of their experience (Hadjikhani et al., 2014).

Thus, mentalizing and experience sharing apparently represent two aspects of the same object, i.e., understanding and responding to another person’s internal states, involving different mental systems. Mentalizing ability examines the theory of mind (ToM) capacity by asking subjects to draw explicit inferences about the mental states of other people. Experience sharing is the tendency to take on, resonate with, or “share” the emotions of others and it is often tied to a mechanism known as “internal resonance” (Zaki and Ochsner, 2012).

It is widely accepted that subjects with ASD do not possess a fully functioning ToM; even high functioning adults with ASD may struggle with complex ToM tasks (Ponnet et al., 2004; Fletcher-Watson et al., 2014). However, affective impairments found in people with ASD are mainly related to the cognitive recognition and processing of emotions, rather than to the actual ability to feel emotional distress or concern. The lack of a clear distinction between affective and cognitive empathy has led to an incomplete understanding of the empathic abilities of individuals with ASD. Interestingly, a few studies have formally assessed empathy in individuals with autistic conditions (Dziobek et al., 2008; Jones et al., 2010; Schwenck et al., 2012). Dziobek et al. (2008) showed an impairment in cognitive empathy, but the presence of normal empathetic concern (affective empathy) in adults with Asperger syndrome (AS), based on self-report questionnaires such as the Interpersonal Reactivity Index (Davis, 1980; Rogers et al., 2007) and the Multifaceted Empathy Test (MET; Dziobek et al., 2008).

Moreover, another two studies (Jones et al., 2010; Schwenck et al., 2012) using only paper and pencil measures, have confirmed that ASD is characterized by difficulties in mentalizing ability (cognitive empathy), but not with affective empathy (Lockwood et al., 2013).

The study of social skills in adolescents with ASD is crucial also for the construction of rehabilitation paradigms to improve empathic capacities. For this reason, in this study we investigated the empathic ability in adolescents with ASD compared to controls, using both paper and pencil and computerized measures, divided into mentalizing and experience sharing abilities in accordance with Zaki and Ochsner’s (2012) model, to evaluate the presence of a dissociation between cognitive and affective empathic abilities in this population.

## MATERIALS AND METHODS

The study included 30 participants: 15 adolescents (11 boys and 4 girls, mean age ±SD: 15.11 ± 4.89 years) were affected by ASD and 15 control subjects (10 boys and 5 girls; mean age = 16.50 ± 6.23), were recruited to match the ASD group with respect to age and education.

Autistic spectrum disorder participants were selected by the Reference Regional Centre for Autism, Abruzzo Region Health System, L’Aquila (Italy). The ASD diagnosis were given by experienced clinicians according to the new criteria of the DSM-5 (American Psychiatric Association, 2013). ASD diagnosis of patients was made with Autism Diagnostic Observation Schedule, Second Edition (ADOS-2; Lord et al., 2012).

Socio-demographic and clinical information of all the participants are summarized in **Table 1**. The parents of adolescents provided informed consent to participate in the study.

**Table 1:** Socio-demographic, mentalizing, and sharing experience measure.

	ASD *(n = 15)* mean scores (SD)	Controls *(n = 15)* means scores (SD)	*t (df = 28)*	*p*
Age (years)	15.11 (4.89)	16.50 (6.23)	-0.686	0.498
Gender	11 M, 4 F	10 M, 5 F		
Education (years)	10.45 (2.55)	10.83 (1.85)	-0.668	0.507
Raven’s matrices (in percentiles)	57.27 (26.96)	43.57 (22.30)	1.119	0.279
**Mentalizing measures**
Advanced ToM task	6.69 (4.15)	12.33 (0.77)	-4.626	**0.0001**
BES cognitive subscale	29.27 (4.01)	39.83 (6.57)	-5.154	**0.0001**
**Sharing experience measures**
Emotion attribution task	27.41 (11.67)	46.75 (10.21)	-4.618	**0.001**
Positive emotions	7.57 (2.92)	9.42 (0.51)	-2.149	0.068
Negative emotions	6.24 (1.59)	8.25 (1.81)	-2.803	**0.011**
Eyes Task	14.50 (8.25)	29.92 (8.62)	-3.142	**0.004**
BES-affective subscale	30 (3.46)	32.17 (7.38)	-7.38	0.322

*Mean scores (and SDs) to the psychological tests separately for ASD and control subjects. The significant results are highlighted by bold numbers.*

### MENTALIZING MEASURES

#### First-order false belief test

This task was designed to elicit a response that demonstrated the ability to make inferences about another individual’s mental state, namely, that a character in the story holds a false belief. First order false beliefs require a subject to make an inference about the state of the world. To assess first order ToM two stories were used: The washing machine task (Rowe et al., 2001; Mazza et al., 2007) and the Cigarettes Task (Happé, 1994).

Each subject obtained a score ranging from 0 to 1 for each question. If the subject gave a correct answer to both the first order stories, s/he had a global score for first order ToM equal to 2 (non-casual performance).

#### Advanced Theory of Mind Task

This task is an Italian adaptation of a cognitive task used by Blair and Cipolotti (2000) and proposed in the literature by Happé (1994). The task consists of a short version of 13 vignettes, each accompanied by two questions; the comprehension question “Was it true, what X said?,” and the justification question “Why did X say that?” The 13 story-types included Lie, White Lie, Joke, Pretend, Misunderstanding, Double Bluff, and Contrary Emotion. Each subject obtained a score ranging from 0 to 1 for each question. The maximum score is 13.

#### Basic Empathy Scale–Cognitive Subscale

The Basic Empathy Scale (BES) comprises a total of 20 items (Jolliffe and Farrington, 2006; Albiero et al., 2009). The cognitive empathy subscale (CE subscale, nine items), measures the ability to understand another person’s emotions. Each item (e.g., “I can often understand how people are feeling even before they tell me”) asks participants to express their own degree of agreement on a 5-point, Likert-type scale, ranging from 1 (“strongly disagree”) to 5 (“strongly agree”). The BES has demonstrated a good validity (Jolliffe and Farrington, 2006; Albiero et al., 2009). Cronbach’s a coefficient was calculated to examine the internal consistency of the scale, considered globally and in its two dimensions, as yielded by the confirmatory factor analysis. The results showed satisfactory internal consistency for both the scale and its subscales, given that the global scale α coefficient was 0.87 and cognitive subscale α values was 0.74 (Albiero et al., 2009).

### EXPERIENCE SHARING MEASURES

*The Eyes Task* is a revised version of the “Reading the Mind in the Eyes Test” (Baron-Cohen et al., 2001). In brief, participants are given 36 photographs depicting the ocular area in an equal number of different actors and actresses. At each corner of every photo, four complex mental state descriptors, e.g., dispirited, bored, are printed, only one of which (the target word) correctly identifies the depicted person’s mental state, while the others are included as foils. The test is scored by totaling the number of items (photographs) correctly identified by the participant; therefore, the maximum total score is 36. In the Italian version the internal consistency (Cronbach’s alpha) was 0.605. Test–retest reliability for the Eyes test, as measured by intraclass correlation coefficient, was 0.833 (95% confidence interval = 0.745 – 0.902).

The study of Vellante et al. (2013) confirms the validity of the Eyes test. Both internal consistency and test–retest stability were good for the Italian version of the Eyes test.

In the *Emotion Attribution Task* (Blair and Cipolotti, 2000). This task assessed ability to represent the emotions of others. In this task, the participant was presented with 58 short stories describing an emotional situation and was required to provide an emotion describing how the main character might feel in that situation. The sentences were designed to elicit attributions of positive and negative emotions. The task was scored according to the number of correct attributions. For this test as well, validation studies are lacking (Mazza et al., 2007).

*The Basic Empathy Scale-Affective subscale* (AE subscale, 11 items): measuring emotional congruence with another person’s emotions. Example items included “I get caught up in other people’s feelings easily.” Each item asks participants to express their own degree of agreement on a 5-point, Likert-type scale, ranging from 1 (“strongly disagree”) to 5 (“strongly agree”). Cronbach’s alpha was 0.86 (Albiero et al., 2009).

### COGNITIVE AND AFFECTIVE EMPATHY MEASURES

#### Multifaceted Empathy Test

To assess empathy multi-dimensionally, we administered the MET (Dziobek et al., 2008), a measure of empathy that allows separate assessments of cognitive and affective aspects of empathic functioning. This test consists of a series of photographs that depict people in emotionally charged situations. In these pictures, taken from the International Affective Picture System (IAPS; Lang, 1980), the stimuli show individuals feeling different emotions: positive emotions (25 pictures that include emotions such as happiness, positive surprise), negative emotions (25 pictures that include emotions such as sadness, anger, disappointment).

Positive and negative emotions were presented in random order. All the stimuli were displayed on a black screen. For each picture the subjects were required to infer the emotional states of the individuals shown in the image by selecting one of four emotional state descriptors (cognitive empathy). To assess affective empathy, subjects rate their level of empathic concern for the individuals displayed in the images on a 9-point Likert scale.

### STATISTICAL METHODS

Mann–Whitney *U* test was used to analyse the level of significance of participants’ scores on First-order false belief task.

*T*-test analysis was used to test significant differences between groups (ASD and control group) in socio-demographic, mentalizing (Advanced ToM and BES-cognitive subscale) and experience sharing measures (Eyes Task, emotion attribution task, and BES-affective subscale).

To evaluate the difference in MET performance between two groups, a 2 × 4 repeated measure design was used. The assumption of normality of the outcome variable was assessed carrying out a Kolmogorov–Smirnov non-parametric test. Restricted maximum likelihood estimation (REML) and an unstructured correlation have been used. Marginal effects have been calculated to getting an estimation of the way the presence of ASD affects the scores of each model predictor. The overall statistical significance of the model has been set at 0.05 level.

The Statistical Package for the Social Sciences (SPSS) software (version 22; SPSS Inc, Chicago, IL, USA) was used for calculating these statistics.

## RESULTS

### MENTALIZING MEASURES

The ASD group showed lower scores compared to the controls in the Advanced ToM Task (*T*_1,28_ = -4.626; *p* = 0.0001), and in BES cognitive subscale (*T*_1,28_ = -5.154; *p* = 0.0001).

In the First-order false belief task the groups differed significantly on the non-parametric Mann–Whitney *U* test (*U* = 86.5; *Z* = -5.44; *p* = 0.0001). The percentage of correct scores in Washing Machine and Cigarette Task was 25.8 and 45.2% for ASD, whereas 100 and 80.8% for the controls, respectively.

Mentalizing performance scores (means and SD) are reported in **Table 1**.

### EXPERIENCE SHARING MEASURES

Adolescents with ASD showed lower scores compared to the control group in the Emotion Attribution Task total score (*T*_1,28_ = -4.618; *p* = 0.001), with a significant difference in negative emotions (*T*_1,28_ = -2.803; *p* = 0.011), but not in positive emotions (*T*_1,28_ = -2.149; *p* = 0.068). Adolescents with ASD also showed lower scores compared to the control group in the Eyes Task (*T*_1,28_ = -3.142; *p* = 0.004), but no significant differences in BES affective subscale (*T*_1,28_ = -7.38; *p* = 0.322) were found.

Experience sharing performance scores (means and SD) are reported in **Table 1**.

### AFFECTIVE AND COGNITIVE EMPATHY MEASURES

#### Multifaceted Empathy Test

Normalized MET data were analyzed by a linear mixed model for repeated measure design with REML. Analysis showed a significant group effect (*z* = -1.18 ± 0.18; *p* < 0.05).

Marginal effects analysis showed statistically significant interaction between ASD and positive cognitive empathy (marginal effect = -0.72 ± 0.18; *p* < 0.05) and between ASD and negative cognitive empathy (marginal effect = -072 ± 0.19; *p* < 0.05).

**FIGURE 1:**
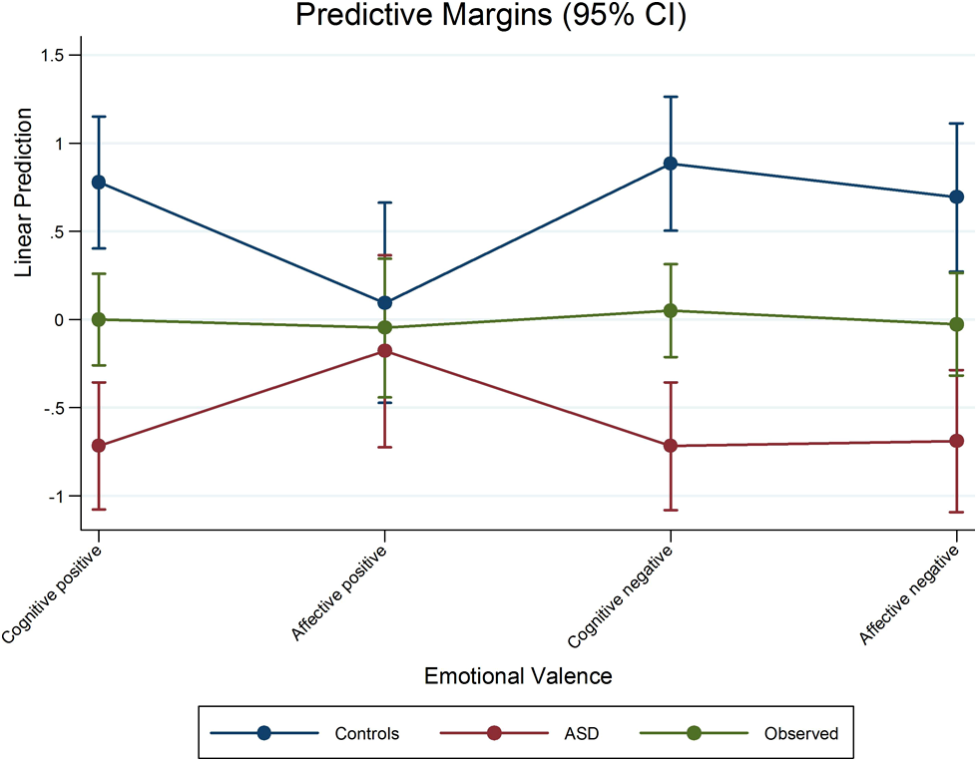
**Marginal effects of the scores of adolescents with ASD and Controls**.

Results showed no significant difference between groups in positive affective empathy (marginal effect = -0.18 ± 0.21; *p* = 0.519). On the other hand, ASD and controls differed in negative affective empathy (marginal effect = -0.69 ± 0.22; *p* < 0.05; see **Table 2**; **Figure 1**).

**Table 2:** Marginal effects of the linear mixed model for repeated measure design with restricted maximum likelihood estimation (REML) of two groups.

Group and group × emotion interaction				
**Subjects**	**Group effect**	**Marginal effect**	**SE**	***p***
Controls		0.61	0.14	**0.001**
ASD		-0.58	0.13	**0.001**

	**Group emotion interaction**			

Controls	Positive cognitive	0.78	0.19	**0.001**
	Negative cognitive	0.88	0.19	**0.001**
	Positive affective	0.09	0.29	0.75
	Negative affective	0.69	0.21	**0.001**
ASD	Positive cognitive	-0.72	0.18	**0.001**
	Negative cognitive	-0.72	0.19	**0.001**
	Positive affective	-0.18	0.28	0.52
	Negative affective	-0.69	0.21	**0.001**

*The significant results are highlighted by bold numbers.*

## DISCUSSION

The aim of this study was to investigate the empathy dimensions in a sample of adolescents with ASD. Specifically, in our research, we examined the empathic abilities in an ASD group compared to normal controls, using a variety of assessment instruments, both paper and pencil and a computerized task. Our data show that adolescents with ASD have a deficit in the cognitive empathy dimension, but do not differ from controls in the affective empathy dimension when other people express emotions with positive valence. Their difficulty in empathizing with the emotional experience of other people is linked to sharing of emotions with negative valence.

Specifically, the results obtained in the paper and pencil measures that investigate mentalizing abilities reveal that the adolescents with ASD hardly interpret other people’s mental states (First-order false belief and Advanced ToM Task) compared to controls. The ASD group also have trouble understanding the meaning of what other people are saying and doing, and they typically struggle to take the other person’s perspective (BES-cognitive subscale).

The evaluation of mentalizing ability through false belief tasks is a key element in investigating the mentalizing skills in individuals with ASD. Therefore, these data confirm that ToM is a core deficit in ASD (Fletcher-Watson et al., 2014; Lai et al., 2014), which links both to precursor skills, such as joint attention and emotion recognition, and to subsequent abilities such as creating friendship and social inclusion. Instead, regarding the sharing experience measures (involving affective empathy, shared self-other representations and emotional contagion), adolescents with ASD were able to empathize with the emotional experience of other people when the latter expressed emotion with a positive valence. In contrast, they showed a deficit in sharing negative emotions. Moreover, the ASD group were unable to share other people’s emotions by observing their ocular region (Eyes Task).

The results obtained in paper and pencil measures were confirmed by the computerized empathy task (MET, Dziobek et al., 2008).

The analysis of affective and cognitive empathy measures evaluated through the MET, showed significant differences between the adolescents with ASD and the control group in the cognitive empathy dimension both when they had to understand and recognize positive and negative emotions.

The cognitive empathic deficits of individuals with ASD could be due to a marked deficit in the ability to understand and explain the mental/emotional states of other people (Jones et al., 2010; Hirvela and Helkama, 2011; Samson et al., 2012; Schwenck et al., 2012; Lockwood et al., 2013).

As far as affective empathy is concerned, the ASD group do not show difficulties in the degree of empathic concern when the emotion is positive, whereas the difficulty is present when observing emotional images with negative valence. The adolescents with ASD feel aroused and involved when others experience positive emotions like the healthy subjects do. Therefore, our results obtained in both measures (paper and pencil and the computerized task) suggest that the ASD subjects showed a difficulty in cognitively identifying the mental state of other people, regardless of the different emotions to which they had to respond; on the other hand, the deficit in affective empathy is linked to emotional valence.

Several studies suggest that the processing of negative emotions is most difficult for individuals with autism (Howard et al., 2000; Ashwin et al., 2006; Corden et al., 2008; Wallace et al., 2008; Humphreys et al., 2013). The role of emotion in autism is still being debated. Ashwin et al. (2006) consider the difficulty of processing negative emotions in subjects with ASD to be linked to an atypical function and structure of the amygdala. In their study people with ASD were less accurate on the emotion recognition task compared to controls, but only for the negative basic emotions. This was discussed in the light of similar findings from people with damage to the amygdala. Based on our results, we assume that the impairment of experience sharing or affective empathy in adolescents with ASD is linked to their poor shared self-other representations of negative emotions.

Blair (2008) has proposed that one of the key processes underpinning functional affective empathy is the recognition of other people’s distress cues (i.e., fear and sadness). Past studies (Howard et al., 2000; Blair, 2008) have shown that children and adolescents with psychopathic tendencies have difficulties in recognizing negative facial and vocal expressions. Thus, it is not possible to speak of impairment of the affective empathy dimension in adolescents with ASD without considering the type of emotion to which the subject responds. Emotional contagion for negative emotions of other people (like sadness, distress, suffering, anger) is important for adaptive social behavior. The lack of sharing experience when other people have negative emotions, leads to a failure of appropriate empathic behavior in adolescents with ASD.

Our results are important for the development of rehabilitation interventions that help these individuals to improve their social skills.

These results are in agreement with recent literature (Jones et al., 2010; Baron-Cohen, 2011; Cox et al., 2012; Schwenck et al., 2012; Lockwood et al., 2013). In particular, Baron-Cohen (2011) shows that cognitive empathy is impaired but affective empathy is not, in individuals with autism. On the contrary, in other psychological conditions, such as psychopathic personality disorder (borderline personality disorder, narcissism, psychopathy), an intact cognitive empathy and impaired affective empathy are present (Baron-Cohen, 2011). The lack of affective empathy, but not of cognitive empathy, seems be an important factor to promote violent and aggressive behaviors.

In conclusion, empathy is a multidimensional construct and requires three abilities: first, the recognition of emotions in oneself and other people via facial expressions, shown by the gaze or behavior; second, the sharing of emotional states with others, i.e., the ability to experience similar emotions to other people while being conscious that this is a simulation of the emotional feeling and it is not one’s own emotion (Derntl et al., 2010) and finally to take the perspective of another person, though the distinction between one’s self and other people remains intact (Decety and Jackson, 2004). For this reason, it is important to use more instruments that allow us to capture all aspects of empathy. Our approach enabled a more detailed analysis of these empathic competencies, also considering the role of emotions in the empathic construct. We believe that this dissociation in cognitive and affective empathy is of importance for several psychiatric conditions which show the empathic ability impairment, such as autism spectrum disorder but also schizophrenia (Fujino et al., 2014) or post-traumatic stress disorder (Mazza et al., 2013). Replication with a larger sample of ASD subjects will be necessary to confirm the present findings.

## AUTHOR CONTRIBUTIONS

Monica Mazza and Marco Valenti designed research; Maria C. Pino, Melania Mariano, and Daniela Tempesta collected data; Monica Mazza, Francesco Masedu, and Domenico De Berardis analyzed the data; Monica Mazza, Maria C. Pino, Michele Ferrara, and Marco Valenti wrote paper.

## Conflict of Interest Statement

The authors declare that the research was conducted in the absence of any commercial or financial relationships that could be construed as a potential conflict of interest.
